# A Review on Liquid Chromatography-Tandem Mass Spectrometry Methods for Rapid Quantification of Oncology Drugs

**DOI:** 10.3390/pharmaceutics10040221

**Published:** 2018-11-08

**Authors:** Andrea Li-Ann Wong, Xiaoqiang Xiang, Pei Shi Ong, Ee Qin Ying Mitchell, Nicholas Syn, Ian Wee, Alan Prem Kumar, Wei Peng Yong, Gautam Sethi, Boon Cher Goh, Paul Chi-Lui Ho, Lingzhi Wang

**Affiliations:** 1 Cancer Science Institute of Singapore, National University of Singapore, Singapore 117599, Singapore; andrea_la_wong@nuhs.edu.sg (A.L.-A.W.); nicholassyn@gmail.com (N.S.); ianwee180894@outlook.com (I.W.); csiapk@nus.edu.sg (A.P.K.); wei_peng_yong@nuhs.edu.sg (W.P.Y.); 2 Department of Haematology-Oncology, National University Health System, Singapore 119228, Singapore; 3 School of Pharmacy, Fudan University, Shanghai 201203, China; xiangxq@fudan.edu.cn; 4 Department of Pharmacy, National University of Singapore, Singapore 117543, Singapore; phaops@nus.edu.sg (P.S.O.); mitchell.ee.q.y@sgh.com.sg (E.Q.Y.M.); 5 Department of Pharmacology, Yong Loo Lin School of Medicine, Singapore 117597, Singapore; phcgs@nus.edu.sg

**Keywords:** LC-MS/MS, rapid, quantification, oncology drugs

## Abstract

In the last decade, the tremendous improvement in the sensitivity and also affordability of liquid chromatography-tandem mass spectrometry (LC-MS/MS) has revolutionized its application in pharmaceutical analysis, resulting in widespread employment of LC-MS/MS in determining pharmaceutical compounds, including anticancer drugs in pharmaceutical research and also industries. Currently, LC-MS/MS has been widely used to quantify small molecule oncology drugs in various biological matrices to support preclinical and clinical pharmacokinetic studies in R&D of oncology drugs. This mini-review article will describe the state-of-the-art LC-MS/MS and its application in rapid quantification of small molecule anticancer drugs. In addition, efforts have also been made in this review to address several key aspects in the development of rapid LC-MS/MS methods, including sample preparation, chromatographic separation, and matrix effect evaluation.

## 1. Introduction

**Figure 1:**
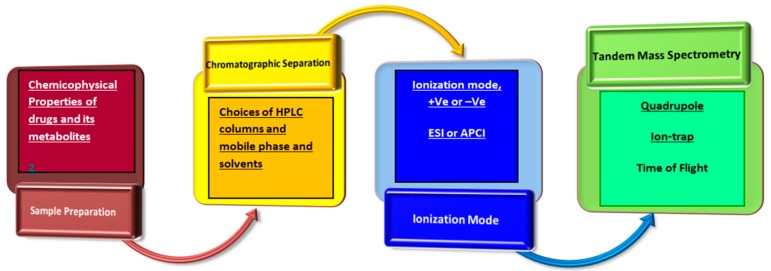
Pipeline for conducting bioanalysis using liquid chromatography-tandem mass spectrometry.

Cancer has a major impact on global health as it is the second leading cause of death. According to the World Cancer Report 2014, the number of new cancer cases is expected to rise by about 70% over the next two decades [1]. This is due to the rapidly ageing population, unhealthy lifestyles, and environmental pollutants, which could consist of carcinogens that can be present in the air, water, and soil, as well as in food as additives or contaminants. Early diagnosis of cancer allows timely treatment of the disease. Unfortunately, the early detection of cancer is still in its infancy as the progress in developing improved early diagnostics and screening tests has been inadequate. For instance, close to 70% of patients with lung cancer present with locally advanced or metastatic disease at the time of diagnosis. Therefore, surgical resection, the single most consistent and successful option for cure, cannot be performed [2]. This thereby makes chemotherapy an important treatment option against cancer. Anticancer drugs are characterized by a narrow therapeutic window. Hence, it is important to understand and manage the inter-individual variability of drug exposure through genotyping/phenotyping and therapeutic drug monitoring (TDM) of anticancer drugs in the clinical setting. In the pharmaceutical industry, the current challenges in the development of anticancer drugs include the significant time and cost required for the preclinical and clinical testing of the new chemical entities (NCEs), and the low success rates. To overcome these obstacles, the pharmaceutical industry has been increasing its effort to improve the efficiency in the processes of drug discovery and development. This is in line with the objective of reducing the attrition rate of NCEs at later stages of the anticancer drug development pipeline, especially in clinical trials as by that stage a large portion of the cost of developing an oncology drug would have been incurred. According to a review article, two major underlying reasons accounting for drug attrition were identified [3]. The first one is due to poor efficacy, which can be overcome by developing more predictive animal models in the preclinical phase. The second crucial factor is owing to poor pharmacokinetics (PK). To overcome the latter hurdle, a high throughput preclinical screening PK approach should be developed and validated. However, the success of PK analysis is highly dependent on the availability of rapid and sensitive bioanalytical assays for quantification of drugs in biological samples. Therefore, rapid and sensitive quantification of anticancer drugs in various biological matrices is urgently needed to discover novel and effective chemotherapeutic agents against various cancers. Liquid chromatography-tandem mass spectrometry (LC-MS/MS) offers superior specificity and sensitivity for compounds without natural chromophores or fluorophores. Hence, this highly sensitive and specific platform has been widely applied in investigating pharmacokinetic properties of novel anticancer drugs in their R&D in preclinical studies as well as in clinical trials. The workflow of LC-MS/MS proposed for bioanalytical method development is shown in Figure 1. The success for developing rapid and sensitive analytical methods is dependent on appropriate sample preparation, fast chromatographic separation to achieve symmetrical peaks, and efficient ionization.

The purpose of this review is to summarize the rapid LC-MS/MS methods for quantification of oncology drugs or potential anticancer compounds published in PubMed and Web of Science databases. In addition, important considerations regarding several crucial aspects in developing rapid LC-MS/MS methods, such as sample preparation, chromatographic separation, matrix effect evaluation, and internal standard selection will be discussed.

## 2. Method of Literature Search

The literature search was conducted using the PubMed and Web of Science databases subscribed to by the National University of Singapore. The title phrase “rapid” or “fast” AND title or abstract phrase “LC-MS/MS” AND title or abstract phrase “cancer” were searched. The articles were then screened for their relevance to rapid quantification of anticancer drugs by LC-MS/MS. No date limitations were applied, and all articles retrieved were dated up to 30 May 2018. Additional relevant literature that was cited in the retrieved articles has also been reviewed. The scope of the review is limited to English language articles only.

## 3. Results and Discussion

Based on the search method described, 72 papers have been found to be relevant to rapid quantification of anticancer drugs using LC-MS/MS methods. All of them have been classified into two categories based on the number of analytes. Forty-nine publications involved analyzing one drug (n = 1) and the remaining 23 involved analyzing multiple drugs/metabolites (n ≥ 2) are summarized in Table 1 [4,5,6,7,8,9,10,11,12,13,14,15,16,17,18,19,20,21,22,23,24,25,26,27,28,29,30,31,32,33,34,35,36,37,38,39,40,41,42,43,44,45,46,47,48,49,50,51,52] and Table 2 [53,54,55,56,57,58,59,60,61,62,63,64,65,66,67,68,69,70,71,72,73,74,75], respectively.

Currently, the unpreceded selectivity and continuously increasing sensitivity of LC-MS/MS have made it a particularly powerful and well established analytical technique to achieve rapid quantitation of anticancer drugs/metabolites in a very small volume of biological samples without tedious chromatographic separation and complicated sample preparation before mass signal detection. Some considerations in developing a LC-MS/MS for rapid quantitation of anticancer drugs will be further elaborated as follows.

### 3.1. Sample Preparation

**Figure 2:**
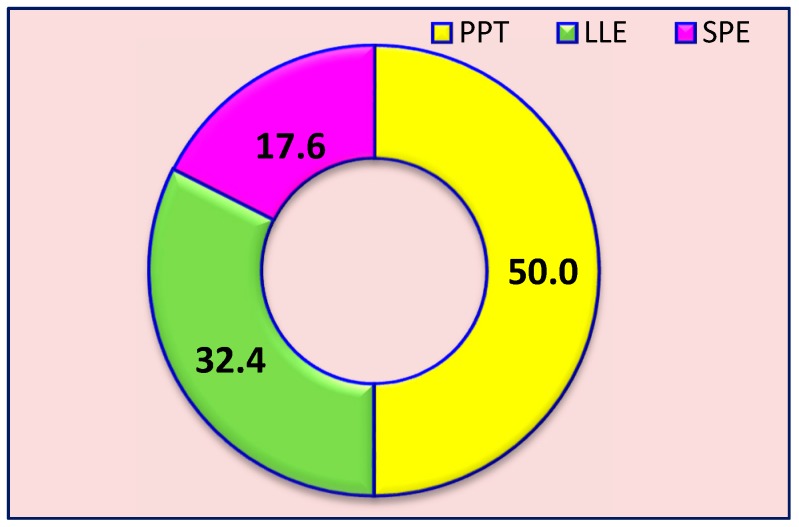
Distribution of sample preparation assays. PPT: protein precipitation; LLE: liquid-liquid extraction; SPE: solid phase extraction.

Efficient sample preparation to avoid severe signal suppression due to the matrix effect is the first key step in achieving high sensitivity and specificity of rapid LC-MS/MS methods. Through sample preparation, we aim to isolate the target drugs/metabolites from the various biological matrices which contain a variety of endogenous components such as proteins, carbohydrates, salts, and lipids, etc. In general, there are three sample preparation methods for purifying the biological samples before injection into the MS/MS analyzer for quantitation of the target analyte(s). These sample preparation procedures can be accomplished through solid-phase extraction (SPE), liquid-liquid extraction (LLE), or protein precipitation (PPT). The results of all the 72 papers reviewed here suggested that PPT accounted for half of them (50%), followed by LLE (32.4%), and then SPE (17.6%), as shown in Figure 2.

SPE makes use of the affinity of solutes dissolved or suspended in a liquid (known as the mobile phase) to a solid matrix when the sample solution or suspension is passed through a cartridge packed with solid matrix, known as the stationary phase. During the process, the undesired endogenous components as well as the exogenous interfering compounds generated during the sample preparation process (e.g., plasticizers released from plastic tubes) have less affinity for the stationary phase. They readily pass through the cartridge leaving the analytes behind in the cartridge. After that, the analytes can be washed out from the cartridge using a different solvent in which the analytes have higher solubility. This process can remove interfering compounds efficiently through optimizing the types of cartridges and solvents used. In addition, it also allows for the enrichment of analytes when very low levels of drugs/metabolites are available to be quantified (e.g., in the situation involving microdosing of anticancer drugs in clinical trials). However, SPE is a labor-intensive process due to its complex procedure including column conditioning, sample loading, washing, and eluting, followed by evaporation of the eluent. This might account for only 18.1% of 72 papers in which the SPE was adopted. In addition, SPE cartridges are more expensive than the small volume of solvent used in LLE and PPT. Furthermore, PPT and LLE are more commonly used in quantification of single drugs/compounds. Only 12.2% of papers cited in Table 1 used SPE for sample preparation, but the usage of LLE is 40.8%, which is 2-fold higher than that of SPE. However, SPE was more popularly used in simultaneous quantification of multiple analytes/metabolites than LLE. Based on Table 2, the percentage of SPE was greatly increased to 30.4% with a substantial decline of LLE usage to 13.0%. Nevertheless, application of SPE cartridges is limited when the drug of interest and its metabolites have very different solubility.

LLE is commonly used in the chemistry laboratory and the pharmaceutical industry to separate compounds based on their relative solubility in two different immiscible liquids, which are usually aqueous or biofluid samples, and an organic solvent such as hexane or ethers. It encompasses an extraction of a substance from one liquid into another liquid phase. LLE is especially suited to lipophilic compounds since the analytes transfer readily from the usually aqueous matrix to an organic phase. This procedure is followed by evaporation of the organic phase with OFN (oxygen-free nitrogen). Comparatively, LLE is much simpler and relatively inexpensive compared to SPE. However, it is not suitable for hydrophilic drugs/metabolites, unless derivatization is done (which is commonly used for GC-MS). Hence, LLE is usually used for determination of a single analyte but not suitable for simultaneous quantification of multiple drugs which have significantly different lipophilicity, resulting in large and different recoveries among the analytes.

PPT is the simplest method of sample pre-treatment as it involves only the addition of a precipitating solvent, subsequent vortex, and centrifugation. The more frequently used solvents for PPT include acetonitrile and methanol. The resulting supernatant is then injected into the LC-MS/MS system for analysis. The advantage of PPT is simple, rapid, and inexpensive. In addition, it is suitable for both lipophilic and hydrophilic analytes. This is a very unique feature as compared to SPE and LLE that cannot extract hydrophilic compounds. This unique property of PPT is very important for quantitative analysis of the relatively hydrophilic drugs or for simultaneous determination of lipophilic drugs with both their lipophilic and hydrophilic metabolites (e.g., exemestane and its phase I and phase II metabolites, 17β-2H-exemestane and 17β-2H-exemestane-*O*-glucuronide) [68]. PPT, however, does not always produce very clean extracts, as many matrix constituents can be extracted simultaneously with the analyte. That can interfere with the MS/MS detection. The interference can be particularly serious when the volume of the biological sample is large (>50 µL).

In summary, there was a significant difference in application of these three methods. The number of studies using PPT alone was equivalent to the combined number of studies using LLE and SPE. In addition, the percentage of LLE at 31.9% was much greater than that of SPE at 18.1%. Based on Table 2, SPE played an important role for simultaneous quantification of multiple drugs/compounds. Only PPT can be used as the sample preparation procedure for the simultaneous quantitation of parent drugs and their hydrophilic metabolites. Taken together, PPT is the most widely used method for preparation of biological samples. The major reason is that most of the metabolites are much more hydrophilic than their parent drugs, particularly for Phase II metabolites which are not able to be extracted with LLE and SPE. However, PPT can extract both a parent drug and its metabolites at an equally high recovery. Therefore, PPT becomes the first choice for sample preparation due to the extremely high selectivity of the MS/MS analyzer and its increasingly improved sensitivity, making quantification of analytes in a small micro volume of biological samples (≤10 µL) possible.

### 3.2. Chromatographic Separation

Chromatography is undoubtedly the most important analytical method for identification and quantitation of a drug and its metabolites since 1952, when Archer J.P. Martin and Richard L.M. Synge were jointly awarded the Nobel Prize in Chemistry for their proposed concept of partition chromatography. Based on the concept, various chromatographic techniques and columns have been developed to separate chemicals with only slight differences in partition coefficients between the mobile and stationary phases. Since the second half of the 20th century, liquid chromatography has been widely used in analysis in the pharmaceutical industry for bioanalysis of drugs in preclinical studies and clinical trials. A lot of analytical methods have been published on the determination of various drugs with liquid chromatography coupled with a UV detector. However, this analytical process used for quantification of analytes in biological samples is quite tedious and time consuming due to the poor selectivity of UV detection—a widely used analytical approach for pharmaceutical analysis in the last century. For analysis using liquid chromatography with UV detection, sample preparation is usually very challenging for analytical scientists as endogenous compounds and co-administered drugs have to be removed via sample preparation as much as possible to minimize the background interference in the analysis. The chromatographic run time is usually long, ranging from 30 to 60 min. This is because the target drugs/metabolites have to be chromatographically separated from both endogenous and exogenous interfering compounds prior to detection and measurement. In the mid last century, a revolutionary change in pharmaceutical analysis was made when the LC-MS/MS was invented. In contrast to most LC-UV analytical methods, LC-MS/MS is able to discern the analyte(s) from the matrix components with the presence of other endogenous substances and spiked internal standards, even if they are co-eluted due to the superior selectivity of MS/MS. However, the potential problem of harmful ion suppression or enhancement from the co-eluting peaks still has to be overcome in the process of developing and validating the LC-MS/MS methods for rapid determination of anticancer drugs/metabolites. This will be further elaborated in the later part of this review.

**Figure 3:**
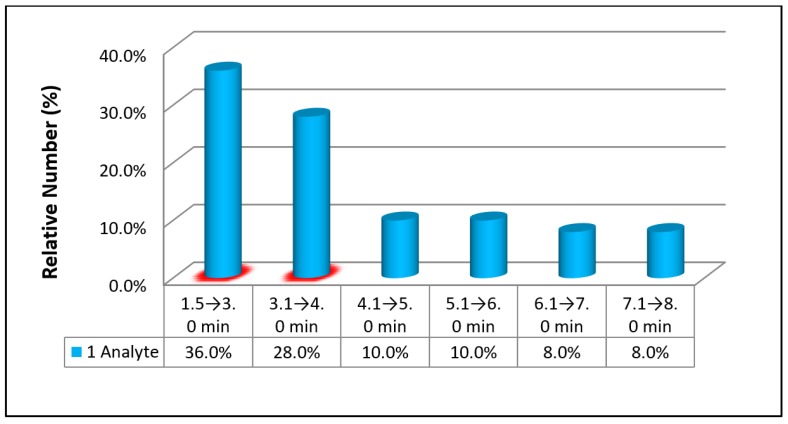
Run time (min) for determination of 1 analyte.

**Figure 4:**
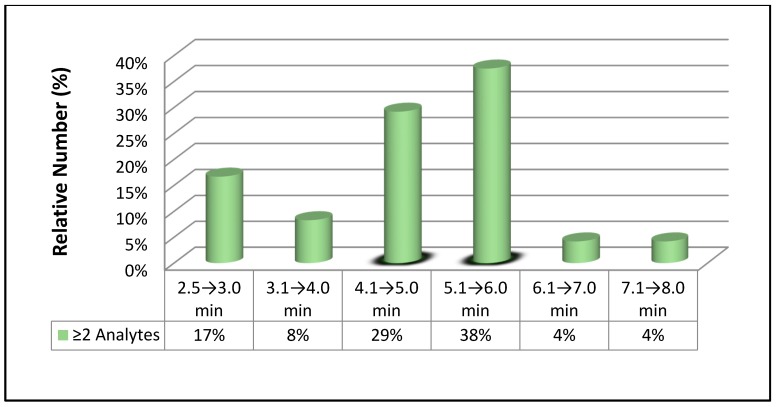
Run time (min) for determination of ≥2 analytes.

The extremely high selectivity and continuously increasing sensitivity of MS/MS lays the foundation for achieving a rapid quantification of analytes in various biological matrices. The run time (RT)—the total time necessary for completing a chromatographic separation—reported in the 72 papers, has been summarized in Table 1 and Table 2. Their distribution was shown in Figure 3 and Figure 4, respectively. Based on Figure 3, the two top percentages of the fastest run times are 36% and 28% (both are shaded) for RT 1.5 → 3.0 min and 3.1 → 4.0, respectively, for the determination of 1 analyte. The combined percentage with these 2 RTs is equal to 64%. Hence, it is reasonable to define a run time of ≤4 min as rapid quantification of one drug in biological matrices. On the other hand, a run time of ≤6 min could be defined as rapid quantification of ≥2 analytes based on Figure 4 in which two top percentages of the fastest run times are 39% and 38% (both are shaded) for RT 4.1 → 5.0 min and 5.1 → 6.0, respectively. This is because as many as 92% of the papers listed in Table 2 reported a RT of ≤6 min for the chromatographic separation of analytes or above. Taken together, the run time to achieve rapid analysis for 1 drug and ≥2 drugs/metabolites are 4 and 6 min, respectively.

The longer run time for determination of ≥2 drugs/metabolites can be explained by the frequent use of gradient elution mode in order to achieve good separation of drugs from the other drugs or their own metabolites. Based on Table 2, as many as 78.3% of the methods for the determination of ≥2 drugs/metabolites adopted gradient elution mode but only 40.8% of the methods for the determination of one drug adopted this elution mode. In general, gradient elution typically takes a longer time in the elution of compounds as the column has to be re-equilibrated back to the starting gradient conditions before reliable retention can be achieved in the subsequent runs. However, the issue on longer run time of gradient elution mode can be circumvented by the use of an ultra-high performance liquid chromatography (UPLC) system. In UPLC, sub-2 µm particles are used in contrast to the standard particle sizes of 3–5 µm used in conventional HPLC columns, resulting in a faster chromatographic analysis [76]. This results in a shorter time for the mobile phase to be re-equilibrated. UPLC coupled with gradient elution works well in determination of multiple analytes. That accounts for 36.6% of all studies, as shown in Table 2. In contrast, gradient elution for UPLC is only used in 6.1% of all studies for analyzing one analyte, as shown in Table 1. For instance, Bouchet et al. reported a well-validated UPLC-MS/MS method for simultaneous determination of nine tyrosine kinase inhibitors within 4 min of run time only [56]. Similarly, Merienne et al. achieved high throughput routine determination of 17 tyrosine kinase inhibitors by another UPLC-MS/MS method [58]. In addition, UPLC separation with gradient mode also improves the peak shapes of the later-eluting compounds and gives chromatographic bands that are more evenly spaced [77].

Contrary to HPLC-UV methods, a baseline chromatographic separation is not needed in LC-MS/MS analysis to elute the target analytes from other interfering compounds during method development, especially for determination of one drug while its metabolites present different mass transitions. However, when a drug and its metabolites are determined simultaneously, the separation between a parent drug and its metabolites is usually necessary as the metabolites, particularly its phase II conjugated metabolites, may have similar fragmentation profiles as the parent drug, leading to inaccurate measurement of the analyte [78].

With reference to the papers listed in Table 2, some authors only indicated the stationary phase and mobile phase conditions used without discussion in detail of the optimization of the chromatographic separation, while the rest reported a multi-factorial optimization on LC column selection (C8 or C18), mobile phase components, and ratios, as well as the flow rates of the mobile phase. Generally, all these optimizations were empirical and not much theoretical explanation was given. Nevertheless, a research group in the US proposed a theory-guided efficient strategy to maximize the speed and resolution in rapid gradient LC-MS/MS analysis [79]. They systematically studied the effect of gradient time, initial and final eluent strength (% organic), and flow rate on the separation resolution and peak capacity in a gradient elution of a mixture of five structurally-related compounds. It was also demonstrated experimentally that increasing flow rate improves both resolution and peak capacity in a rapid gradient method. The results can be well explained mathematically using the linear-solvent-strength (LSS) gradient theory. This further supports our finding that UPLC-coupled gradient elution is an efficient approach for simultaneous quantification of multiple analytes in a short run time (≤6 min). In regard to internal standards, as many as 74.3 of the internal standards from 72 papers are structural analogues even though stable isotopically labelled (SIL) analogues of the analytes are preferred in achieving better quantitative results. The main reasons are (1) not commercially available or (2) too expensive.

### 3.3. Matrix Effects

Although MS/MS has been demonstrated to possess superior selectivity and sensitivity, the signal is often affected significantly by the biological matrix residues. Ion suppression or enhancement remains an inherent problem in LC-MS/MS method development and could be the result of interference of endogenous substances from the biological matrices (e.g., human plasma) or exogenous substances during sample preparation (e.g., polymers from polypropylenetubes) [80]. The alteration of ionization efficiency by the presence of co-eluting substances is called “matrix effects”. These effects are not detectable in the chromatogram but have deleterious impacts on the method’s accuracy and sensitivity. Hence, an assessment of matrix effects is needed according to the European Medicine Agency (EMA) and USA Food and Drug Administration (FDA) guidelines so as to ensure that precision, selectivity, and sensitivity of LC-MS/MS analyses are not compromised [81]. A series of experiments were conducted to explore the mechanism of matrix effects and the authors concluded that the possible reason was due to the result of competition between non-volatile matrix components and analyte ions for access to the droplet surface for transfer to the gas phase [82]. Therefore, application of suitable methods for the evaluation of matrix effects plays an important role in developing and validating a sensitive and robust analytical method for the determination of anticancer drugs/metabolites in biological matrices.

Generally, there are two common methods to assess matrix effects. One is the post-extraction addition method, while the other is the post-column infusion method. In 2003, Matuszewski et al. published a research paper discussing the strategies for the assessment of the matrix effect in quantitative bioanalytical methods based on LC-MS/MS [80]. The matrix effect during validation of analytical methods in biological fluids may be best examined by comparing the MS/MS response (peak areas or peak heights) of an analyte at any given concentration spiked post-extraction into a biological fluid extract (B), to the MS/MS response (A) of the same analyte present in the “neat” mobile phase. The equation of matrix effect (%) can be expressed as follows:

ME (%) = B/A × 100.(1)

**Figure 5:**
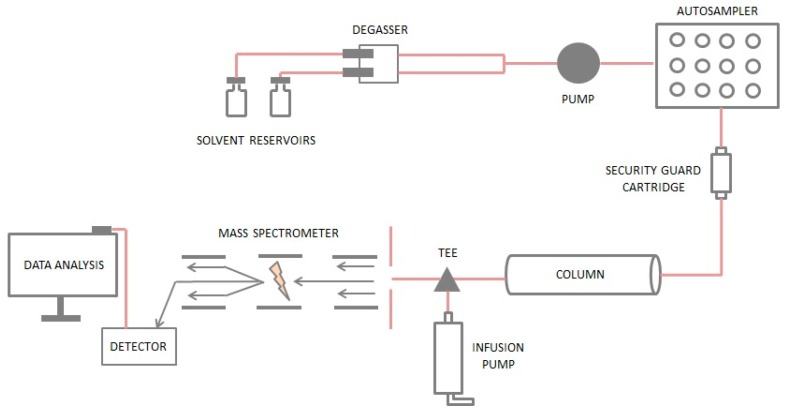
The schematic of the post-column infusion system for evaluation of matrix effects.

A value of 100% indicates that the responses in the “neat” mobile phase and the plasma extracts were the same and no absolute matrix effect was observed. A value of >100% indicates an ionization enhancement and a value of <100% indicates an ionization suppression. The post-extraction addition technique is a quantitative but static approach that only provides information about matrix effects at the point of elution of the analyte. A more dynamic technique for determining matrix effects is the post-column infusion method [82]. The post-column infusion system is schematically represented in Figure 5. An infusion pump was used to deliver a constant flow of analyte at a concentration in the range of quantitation into the chromatographic eluent at a point after the column and before the mass spectrometer ionization source [83]. A sample of extract (without added analyte) was injected under the desired chromatographic conditions and the response from the infused analyte recorded. The post-infusion technique enables the influence of the matrix on analyte response to be investigated over the entire chromatographic run. Nevertheless, the post-infusion approach is a qualitative or semi-quantitative method. It can be used to evaluate the influence of different sample extraction methods, chromatographic conditions such as mobile phase components, and analytical columns on matrix effects.

Due to the critical influence of matrix effects on mass analyzers, matrix effects have to be evaluated systematically during the development of well-validated and rapid LC-MS/MS methods. Among the 72 papers of rapid analytical LC-MS/MS methods for determination of anticancer drugs and their metabolites in Table 1 and Table 2, 11 papers did not mention matrix effects. Two of them were published in 2003 when the impact of matrix effects on the LC-MS/MS methods had not been fully recognized by analytical scientists. Nevertheless, the majority of the studies incorporated in our review (61 out of 72, 84.7%) have reported matrix effects of the analytes in various biological samples during method development and validation. In addition, the matrix effects in all of these 61 papers were evaluated using the post-extraction addition approach. The reasonable explanation is that the post-extraction addition technique is a quantitative approach for the evaluation of matrix effects on the analytes. Based on the quantitative analysis of matrix effects, effective solutions to overcome potential matrix effects or at least minimize the influence of matrix effects on sensitivity and accuracy of the LC-MS/MS methods are needed during method development.

The matrix effect is a common phenomenon in the quantitation of drugs and metabolites in biological matrices using LC-MS/MS. Since the matrix effect could be potentially caused by the influence of co-eluting non-volatile matrix components on the ionization efficiency of the analytes, it can be minimized, avoided, or compensated mainly through optimization of sample preparation, chromatographic separation, and suitable internal standard, respectively. In theory, SPE is an ideal sample preparation assay in which matrix effects can be eliminated efficiently because the analytes can be efficiently isolated from the matrix via suitable SPE columns and elution solutions. However, it is a very tedious and time-consuming process. Comparatively, LLE is a simpler and faster procedure for the preparation of biological samples but the purified samples may still contain some lipophilic endogenous compounds which could potentially affect the quantification of analytes. In such a case, chromatographic separation can be optimized to minimize the resulting matrix effects due to inherent limitation of LLE. As a widely used bio-sample preparation assay, PPT is the most convenient approach, but the purified samples may also be much dirtier than the samples extracted by the SPE or LLE techniques. However, an important fact to note is that increasingly improved sensitivity of LC-MS/MS provides us a good chance to use a minute volume of biological samples, e.g., 5 µL of plasma or serum for analysis. In this scenario, the residue of impurities derived from PPT is negligible in most cases. This is the reason why PPT was adopted as the sample preparation for rapid determination of anticancer drugs/metabolites using a LC-MS/MS platform in the majority of the 72 papers reviewed.

## 4. Conclusions and Perspectives

Rapid liquid chromatography-tandem mass spectrometry plays an important role in both preclinical development and clinical trials. Based on the papers published in English, the assay run times of rapid LC-MS/MS methods for a single analyte and multiple analytes were identified as 4 and 6 min, respectively. With the development of UPLC systems and the availability of more isotopically-labelled internal standards, assay run times for rapid analysis of anticancer drugs/metabolites could be further reduced in order to accelerate drug development.

In the preparation of biological samples, PPT is widely applied as it is the simplest sample preparation approach and can be used to quantify both hydrophilic and lipophilic compounds simultaneously, thereby making it the most popular method compared to SPE and LLE. Structural analogues are mostly used as internal standards, given the consideration of costs and availability. In the future, great effort should be made to establish the principles in selection of appropriate internal standards, which are chosen mainly based on a trial and error approach.

Currently, LC-MS/MS has been widely used to investigate pharmacokinetics of oncology drugs to support early phase clinical trials and determine potential drug–drug interactions. The advantage in using LC-MS/MS is its super sensitivity and specificity, which makes it a powerful tool for clinical therapeutic monitoring of oncology drugs.

## Figures and Tables

**Table 1:** LC-MS/MS methods for determination of one drug.

Analyte(s)	Indication	Matrices	Prep	Solid Phase	E-M	Interf	IS	ME (%)	RT (min)	LLOQ	Ref.
Nimorazole	Radiosensitizer	r-plasma	PPT	C18 (50 × 4.6 mm, 2.7 µm)	ISO	ESI(+)	AN	NEG	1.5	0.25 ng/mL	[4]
JI-101	Multi-kinase inhibitor	h-plasma h-urine	SPE	C18 (50 × 2.1 mm, 5 µm)	ISO	ESI(+)	AN	98	2.0	1.57 ng/mL 0.97 ng/mL	[5]
LBH589	HDAC inhibitor	m-plasma m-tissue	LLE	C18 (50 × 2.1 mm, 1.7 µm) UPLC	ISO	ESI(+)	AN	NEG	2.0	2.5 ng/mL 35.7 ng/mg	[6]
Vinorelbine	Vinca alkaloid	h-plasma	LLE	C18 (50 × 2.1 mm, 3 µm)	ISO	ESI(+)	AN	95.8–106.7	2.0	0.1 ng/mL	[7]
Cerivastatin	Inhibitor of HMG-CoA reductase	h-plasma	LLE	C18 (100 × 3 mm, 3.5 µm)	ISO	ESI(+)	AN	NEG	2.0	0.01 ng/mL	[8]
Osimertinib	Tyrosine kinase inhibitor	r-plasma	PPT	C18 (50 × 2.1 mm, 3 µm)	GRA	ESI(+)	AN	90.1–97.3	2.5	1 ng/mL	[9]
Anastrazole	Aromatase inhibitor	h-plasma	SPE	C18 (50 × 4.6 mm, 5 μm) UPLC	ISO	ESI(+)	AN	97.5	2.5	0.3 ng/mL	[10]
SZ-685C	Marine anticancer agent	r-plasma	LLE	C18 (100 × 2.1 mm, 3 µm)	ISO	ESI(−)	AN	94.3	2.5	5 ng/mL	[11]
CLR1401	Anticancer candidate	r-plasma	LLE	C18 (50 × 3.0 mm, 5 µm)	GRA	ESI(+)	IL	80.0–86.0	2.8	2 ng/mL	[12]
Veliparib (ABT-888)	PARP-1 & 2 inhibitor	h-plasma	PPT	C18 (100 × 2.1 mm, 3 µm)	ISO	ESI(+)	AN	UNK	3.0	5 nmol/L	[13]
Docetaxel	Anticancer drug	h-plasma	LLE	C8 (50 × 2.1 mm, 5 µm)	ISO	ESI(+)	AN	UNK	3.0	5 ng/mL	[14]
Aucubin	Natural compound	r-plasma	PPT	Diamonsil C18(2)	ISO	ESI(+)	AN	90.8–91.0	3.0	10 ng/mL	[15]
HCQ	Inhibitor of autophagy	h-blood	PPT	C8 (50 × 2.1 mm, 5 µm)	ISO	ESI(+)	IL	93.0–100.6	3.0	5 ng/mL	[16]
Sunitinib	Tyrosine kinase inhibitor	h-plasma	LLE	C18 (50 × 2.1 mm, 3.5 µm)	ISO	ESI(+)	AN	UNK	3.0	0.2 ng/mL	[17]
DZNep	Methylation inhibitor	m-plasma	LLE	HILIC (100 × 2.1 mm, 1.7 µm) UPLC	GRA	ESI(+)	AN	84–87	3.0	5 ng/mL	[18]
SN-38	Anticancer drug	h-plasma	PPT	C18 (50 × 2.0 mm, 4 µm)	GRA	ESI(+)	AN	UNK	3.0	0.05 ng/mL	[19]
Vincristine	Anticancer drug	h-plasma	PPT	C18 (50 × 2.1 mm, 5 µm)	ISO	APCI(+)	AN	UNK	3.0	0.1 ng/mL	[20]
MS-275	HDAC inhibitor	h-plasma	LLE	C18(50 × 2.1 mm, 3.5 μm)	GRA	ESI(+)	AN	UNK	3.0	0.5 ng/mL	[21]
trans-resveratrol	Natural compound	m-plasma m-brain	LLE	C18 (100 × 1 mm, 5 μm)	ISO	ESI(−)	AN	93.8–100.6	3.0	5 ng/mL	[22]
ZD6474	Tyrosine kinase inhibitor	h-plasma h-fluid	LLE	C18 (50 × 2.1 mm, 2.6 µm)	ISO	ESI(+)	IL	98.0	3.0	0.25 ng/mL 0.25 ng/mL	[23]
Crizotinib	Tyrosine kinase inhibitor	r-plasma	PPT	Zorbax XDB C18 (2.1 × 50 mm, 3.5 μm)	GRA	ESI(+)	AN	94.3–96.2	3.5	1 ng/mL	[24]
cabozantinib	Tyrosine kinase inhibitor	r-plasma	LLE	C18 (50 × 2 mm, 5 μm)	ISO	ESI(+)	AN	105–115	3.5	0.5 ng/mL	[25]
KPS-A	Natural compound	r-plasma	PPT	C18 (2.1 × 50 mm, 3.5 μm)	GRA	ESI(+)	AN	93–96	3.5	0.5 ng/mL	[26]
Clofarabine triphosphate	Metabolite of clofarabine	h-PBMC	PPT	CN (100 × 4.6 mm, 5 μm)	GRA	ESI(+)	AN	91–105	3.5	1.25 ng/10^7^ cells	[27]
Paclitaxel	Antimicrotubule agent	r-plasma r-tissue	LLE	C8 (50 × 2.1 mm, 5 μm)	ISO	ESI(+)	IL	70.9–82.7	3.5	0.5 ng/mL 1.5 ng/g	[28]
EDL-155	Anticancer agent	r-plasma	PPT	C8 (50 × 2.1 mm, 3.5 μm)	GRA	ESI(+)	AN	98.6	3.5	0.1 ng/mL	[29]
Henatinib	Kinase inhibitor	h-plasma h-urine	PPT	C18 (50 × 2.1 mm, 2.5 μm)	ISO	ESI(+)	AN	90.5–100.9	3.5	0.1 ng/mL 1 ng/mL	[30]
Ceritinib	ALK inhibitor	h-plasma h-brain	PPT	C18 (50 × 2.1 mm, 2.7 μm)	GRA	ESI(+)	IL	92–109	3.6	1 ng/mL	[31]
Methergine	chemosensitizer for cancer	h-plasma	LLE	C18 (100 × 2.1 mm, 2.7 μm)	ISO	ESI(+)	AN	61–66	4.0	0.025 ng/mL	[32]
Letrozole	Aromatase inhibitor	h-plasma	SPE	C18 (100 × 2.1 mm, 3.5 μm)	ISO	ESI(+)	AN	NEG	4.0	0.25 ng/mL	[33]
Deacetyl mycoepoxydiene	Marine anticancer agent	r-plasma	PPT	C18 (150 × 2.1 mm, 5 μm)	ISO	ESI(+)	AN	95.5–97.8	4.0	5 ng/mL	[34]
Sorafenib	Kinase inhibitor	h-plasma	PPT	SymmetryShield RP8 (50 × 2.1 mm, 3.5 μm) (0.1% FA:ACN)	ISO	ESI(+)	IL	98.6	4.0	5 ng/mL	[35]
QBH-196	c-Met tyrosine kinase inhibitor	r-plasma	LLE	C18 (50 × 2.1 mm, 2.6 µm)	GRA	ESI(+)	AN	80–115	4.0	8 ng/mL	[36]
Fenretinide	Chemopreventive agent	m-plasma	PPT	C18 (50 × 2.1 mm, 5 μm)	GRA	APCI(+)	AN	100.8–108.7	4.5	0.5 ng/mL	[37]
PM01183	Antineoplastic agent	Animal plasma	SPE	C18 (30 × 2.1 mm, 3 μm)	GRA	ESI(+)	IL	88–103	5.0	0.1 ng/mL	[38]
JCC76	Antitumor agent	r-plasma	LLE	C18 (40 × 2.0 mm, 5 μm)	ISO	ESI(−)	AN	90.8–96.9	5.0	0.3 ng/mL	[39]
Megestrol acetate	Hormonal therapy	h-plasma	LLE	C18 (50 × 2.0 mm, 3 µm)	ISO	ESI(+)	AN	92.3–95.8	5.0	1.0 ng/mL	[40]
Berbamine	Natural compound	r-plasma	PPT	C18 (150 × 2.0 mm, 5 μm)	GRA	ESI(+)	AN	97.2–98.5	5.5	1 ng/mL	[41]
Peri-plocymarin	potential anticancer agent	r-plasma r-tissue	LLE	C18 (2.1 × 150 mm, 3.0 μm)	ISO	ESI(+)	AN	95.8–105	6.0	0.5 ng/mL	[42]
ABL	potential anticancer agent	r-plasma	PPT	C18 (50 × 4.6 mm, 3.0 μm)	ISO	ESI(+)	AN	104–108	6.0	1.6 ng/mL	[43]
Cisplatin	Anticancer drug	r-tissue	LLE	C18 (50 × 2.1 mm, 1.8 μm)	ISO	ESI(+)	AN	89–104	6.0	5 ng/mL	[44]
EC-18	Anticancer agent	r-plasma m-plasma	PPT	C18 (150 × 2 mm, 4.0 μm)	GRA	ESI(+)	IL	77.9–89.0	7.0	50 ng/mL	[45]
Z-endoxifen	Anti-estrogen	h-serum	PPT	C18 (150 × 2.1 mm, 2.6 µm)	GRA	ESI(+)	IL	NA	7.0	1 ng/mL	[46]
5-azacytidine	Anticancer agent	h-plasma	SPE	C18 (250 × 2.1 mm, 4 µm)	ISO	ESI(+)	AN	51–55	7.0	5 ng/mL	[47]
RGB-286638	Protein kinase inhibitor	h-plasma h-urine	LLE	C18 (50 × 2.1 mm, 5 µm)	GRA	ESI(+)	IL	146–151	7.0	2 ng/mL 2 ng/mL	[48]
Azurin p28	Anticancer peptide	m-ser	PPT	C18 (100 × 2 mm, 5 µm)	GRA	ESI(+)	AN	UNK	7.5	100 ng/mL	[49]
Apogossypol	Bcl-2 inhibitor	m-plasma	PPT	C18 (100 × 2 mm, 4 µm)	GRA	ESI(+)	AN	UNK	7.5	10 ng/mL	[50]
Methotrexate	Anticancer drug	h-saliva	SPE	C18 (150 × 2.0 mm, 2.2 μm)	GRA	ESI(+)	AN	96–104	8.0	1.0 ng/mL	[51]
CSUOH0901	COX-2 inhibitor	r-plasma	PPT	C18 (50 × 2.0 mm, 5 μm)	GRA	ESI(+)	AN	90.1–94.5	8.0	0.5 ng/mL	[52]

Abbreviations: Prep: sample preparation; S-Ph (M-Ph): Solid phase (Mobile phase); E-M: Elution mode; Interf: Interface; IS: Internal standard; RT: Run time; LLOQ: Lower limit of quantitation; Ref.: Reference number; h: human; m: mouse; r: rat; d: dog; LLE: liquid-liquid extraction; SPE: solid phase extraction; PPT: protein precipitation; ISO: isocratic elution; GRA: gradient elution; AN: analogue internal standard; IL: isotope labeled internal standard; LBH589: Panobinostat; HCQ: Hydroxychloroquine; JCC76: Cyclohexanecarboxylic acid [3-(2,5-dimethyl-benzyloxy)-4-(methanesulfonyl-methyl-amino)-phenyl]-amide; DZNep: 3-Deazaneplanocin A; EDL-155: 1,2,3,4-tetrahydroisoquinoline; ZD6474: vandetanib; PR104: (A: alcohol; H: hydroxylamine; M: amine; G: O-glucuronide); CA4P: combretastatin A4 phosphate; CA4: combretastatin A4; CA4G: combretastatin A4 glucuronide; ABL:1-O-acetylbritannilactone; KPS-A: kalopanaxsaponin A; QBH-196: N1-(3-fluoro-4-{6-methoxy-7-[3-(4-methylpiperidin-1-yl) propoxy] quinolin-4-yloxy}phenyl)-N4-(2,4-difulurobenzylidene) semicarbazided; NA: not available; NEG: negligible matrix effect; UNK: unknown; ME: matrix effect; UPLC: Ultra-Performance Liquid Chromatography.

**Table 2:** LC-MS/MS methods for determination of two or more drugs/metabolites.

Analyte(s)	Indication	Matrices	Prep	S-Ph	E-Mode	Interf	IS	ME (%)	RT (min)	LLOQ	Ref.
Belinostat Panobinostat Rocilinostat Vorinostat	HDAC inhibitor	r-plasma	PPT	C18 (50 × 4.6 mm, 5 μm)	ISO	ESI(+)	AN	No significant ME	2.5	2.9 ng/mL 2.9 ng/mL 1.0 ng/mL 1.0 ng/mL	[53]
CT-707 CT-707M1 CT-707M2	Tyrosine kinase selective inhibitor	h-plasma	SPE	C18 (2.1 × 50 mm, 1.7 μm) UPLC	GRA	ESI(+)	IL	86.9–102	3.0	2 ng/mL 1 ng/mL 1 mg/mL	[54]
Gefitinib O-DMG	EGFR inhibitor	h-plasma	PPT	C18 (150 × 2.1 mm, 5 µm)	ISO	ESI(+)	AN	93.0–103.3; 41.6–50.2	3.0	5 nmol/L	[55]
Sunitinib Gefitinib Norimatinib (met) Imatinib Dasatinib Erlotinib Axitinib Nilotinib Lapatinib Sorafenib	Nine tyrosine kinase inhibitors and one metabolite of Imatinib	h-pls	SPE	C18 (50 × 2.1 mm, 1.7 μm) UPLC	GRA	ESI(+)	IL	96.6 104.5 85.5 85.0 84.5 81.6 113.1 101.8 91.2 107.7	4.0	10 ng/mL 0.1 ng/mL 10 mg/mL 10 ng/mL 0.1 ng/mL 10 mg/mL 0.1 ng/mL 10 ng/mL 10 mg/mL 0.1 mg/mL	[56]
MTX 7-OH-MTX	Anticancer drug	m-plasma m-brain	PPT	C18 (50 × 2.0 mm, 5 µm)	ISO	ESI(+)	IL	88.2–108.8	4.0	3.7 ng/mL 7.4 ng/mL	[57]
17 tyrosine kinase inhibitors	EGFR tyrosine kinase inhibitors	h-plasma	SPE	C18 (5 × 2.1 mm, 1.6 µm) UPLC	GRA	ESI(+)	IL	83.4–116.40	5.0	0.1 ng/mL	[58]
Doxorubicin L-DOX	Anticancer antibiotic	h-plasma	SPE	C18 (50 × 2.1 mm, 5.0 μm)	GRA	ESI(+)	AN IL	95.7–98.9	5.0	3.13 ng/mL 0.15 μg/mL	[59]
Vemurafenib, Dabrafenib Cobimetinib, Trametinib Binimetinib	2 BRAF inhibitors 3 MEK inhibitors	h-plasma	SPE	C18 (100 × 2.1 mm, 5.0 μm) UPLC	GRA	ESI(+)	IL	80.6–115.4	5.0	0.4 ng/mL 1.0 ng/mL 0.5 ng/mL 0.5 ng/mL 0.75 ng/mL	[60]
Thalidomide Lenalidomide Cyclophosphamide Bortezomib Dexamethasone Adriamycin	Anticancer drug	h-serum	SPE	C18 (50 × 2.1 mm, 2.5 μm)	GRA	ESI(+)	AN	89–100 60–64 113–124 103–126 90–92 143–163	5.0	4 ng/mL 2 ng/mL 2 ng/mL 2 ng/mL 2 ng/mL 2 ng/mL	[61]
MG PGG	Natural compounds	r-blood	LLE	C18 (50 × 2.1 mm, 5 µm) UPLC	GRA	ESI(+)	AN	76–87 80–104	5.0	19.5 nmol/L 39 nmol/L	[62]
Allitinib M6 M10	Irreversible inhibitor of the EGFR 1/2	h-plasma	PPT	C18 (50 × 4.6 mm, 1.8 µm)	GRA	ESI(+)	AN	98.7–105.0	5.0	0.3 ng/mL 0.03 ng/mL 0.075 ng/mL	[63]
Gefitinib Erlotinib Afatinib	EGFR tyrosine kinase inhibitors	h-plasma	LLE	C18 (50 × 2.1 mm, 3.5 µm) UPLC	ISO	ESI(+)	AN	UNK	5.0	0.01 nmol/L 0.01 nmol/L 0.05 nmol/L	[64]
CP 4OHCP	Anticancer drug	h-plasma	PPT	C18 (150 × 2.1 mm, 5 μm)	GRA	ESI(+)	IL AN	UNK	6.0	0.2 µg/mL 0.05 µg/mL	[65]
Clofarabine Cytarabine	Anticancer drug	h-plasma	PPT	C18 (150 × 2.0 mm, 4 μm)	GRA	ESI(+)	AN	None	6.0	8 ng/mL 20 ng/mL	[66]
Gefitinib M523595 M537194 M387783 M608236	EGFR tyrosine kinase inhibitor & its metabolites	m-plasma	PPT	C18 (50 × 2.1 mm, 1.8 m)	GRA	ESI(+)	AN	86–112	6.0	1 ng/mL 1 ng/mL 1 ng/mL 1 ng/mL 1 ng/mL 0.5 ng/mL	[67]
Exemestane 17β-2H-EXE 17β-2H-EXE-Glu	Steroidal aromatase inhibitor	h-plasma	PPT	C18 (100 × 2.1 mm, 5 µm)	GRA	ESI(+)	AN	62.2 54.2 33.8	6.0	0.4 ng/mL 0.2 ng/mL 0.2 ng/mL	[68]
CPT-11 SN-38 SN-38G APC NPC	Topoisomerase I inhibitor	h-plasma	PPT	C18 (50 × 2.0 mm, 2.6 µm)	GRA	ESI(+)	AN	91.0	6.0	0.5 ng/mL 0.2 ng/mL 0.5 ng/mL 0.5 ng/mL 0.2 ng/mL	[69]
Sinotecan 7-HEC	Anticancer agent	h-blood	PPT	C8 (150 × 4.6 mm, 5 µm)	GRA	ESI(+)	AN	104–114	6.0	1 ng/mL 0.5 ng/mL	[70]
Letrozole Carbinol carbinol glucuronide	Aromatase inhibitor and its metabolites	h-pls	SPE	C18 (50 × 4.6 mm, 1.8 μm) UPLC	GRA	ESI(+)	IL	30–31 90–100	6.0	20 nmol/L 0.2 nmol/L 2 nmol/L	[71]
PR104 PR-104A PR-104G PR-104H PR-104M	Hypoxia-activated prodrug	h-plasma	PPT	C18 (50 × 2.1 mm, 1.8 µm) UPLC	GRA	ESI(+)	IL	87.4–112.6	6.0	0.1 µmol/L 0.05 µmol/L 0.05 µmol/L 0.025 µmol/L 0.01 µmol/L	[72]
CA4P CA4 CA4-Glu	Antitumor vascular disrupting agent	d-plasma	PPT	C18 (150 × 3.0 mm, 5 μm)	GRA	ESI(+) ESI(+) ESI(−)	AN	NEG	6.0	5 ng/mL	[73]
Paclitaxel Docetaxel Vinblastine Vinorelbine	Regulators of microtubule formation	h-plasma	LLE	C18 (100 × 2.1 mm, 3.5 µm)	ISO	ESI(+)	AN	86.7–102.5	6.0	25 ng/mL 10 ng/mL 10 ng/mL 10 ng/mL	[74]
17AAG 17AG	HSP90 inhibitor	h-plasma	PPT	C18 (50 × 2.1 mm, 5 μm)	GRA	ESI(+)	AN	UNK	7.0	0.5 ng/mL 0.5 ng/mL	[75]

Abbreviations: Prep: sample preparation; S-Ph (M-Ph): Solid phase (Mobile phase); E-mode: Elution mode; Interf: Interface; IS: Internal standard; RT: Run time; LLOQ: Lower limit of quantitation; Ref.: Reference number; h: human; m: mouse; r: rat; d: dog; LLE: liquid-liquid extraction; SPE: solid phase extraction; PPT: protein precipitation; ISO: isocratic elution; GRA: gradient elution; AN: analogue internal standard; IL: isotope labeled internal standard; CP: Cyclophosphamide; 4OHCP: 4-hydroxycyclophosphamide; O-DMG:O-desmethyl gefitinib; MTX: Methotrexate; L-DOX: Liposomal doxorubicin; LBH589: Panobinostat; MG: methyl gallate; PGG: pentagalloyl glucopyranose; 17β-2H-EXE: 17β-hydroxyexemestane; 17β-2H-EXE-Glu: 17β-hydroxyexemestane-17-O-β-D-glucuronide A; 7-HEC: 7-hydroxyethyl-camptothecin; PR104: (A: alcohol; H: hydroxylamine; M: amine; G: O-glucuronide); CA4P: combretastatin A4 phosphate; CA4: combretastatin A4; CA4G: combretastatin A4 glucuronide; 17AAG: 17-(allylamino)-17-demethoxygeldanamycin; 17AG: 17-amino-17-demethoxygeldanamycin; NEG: negligible matrix effect; SIG: significant matrix effect; UNK: unknown.
